# Epithelial Cells and Inflammation in Pulmonary Wound Repair

**DOI:** 10.3390/cells10020339

**Published:** 2021-02-05

**Authors:** Amanda Croasdell Lucchini, Naomi N. Gachanja, Adriano G. Rossi, David A. Dorward, Christopher D. Lucas

**Affiliations:** University of Edinburgh Centre for Inflammation Research, Queen’s Medical Research Institute, Edinburgh Bioquarter, Edinburgh EH16 4TJ, UK; amandaclucchini@gmail.com (A.C.L.); n.gachanja@sms.ed.ac.uk (N.N.G.); a.g.rossi@ed.ac.uk (A.G.R.); david.dorward@ed.ac.uk (D.A.D.)

**Keywords:** epithelium, lung, regeneration, repair, inflammation, injury, resolution

## Abstract

Respiratory diseases are frequently characterised by epithelial injury, airway inflammation, defective tissue repair, and airway remodelling. This may occur in a subacute or chronic context, such as asthma and chronic obstructive pulmonary disease, or occur acutely as in pathogen challenge and acute respiratory distress syndrome (ARDS). Despite the frequent challenge of lung homeostasis, not all pulmonary insults lead to disease. Traditionally thought of as a quiescent organ, emerging evidence highlights that the lung has significant capacity to respond to injury by repairing and replacing damaged cells. This occurs with the appropriate and timely resolution of inflammation and concurrent initiation of tissue repair programmes. Airway epithelial cells are key effectors in lung homeostasis and host defence; continual exposure to pathogens, toxins, and particulate matter challenge homeostasis, requiring robust defence and repair mechanisms. As such, the epithelium is critically involved in the return to homeostasis, orchestrating the resolution of inflammation and initiating tissue repair. This review examines the pivotal role of pulmonary airway epithelial cells in initiating and moderating tissue repair and restitution. We discuss emerging evidence of the interactions between airway epithelial cells and candidate stem or progenitor cells to initiate tissue repair as well as with cells of the innate and adaptive immune systems in driving successful tissue regeneration. Understanding the mechanisms of intercellular communication is rapidly increasing, and a major focus of this review includes the various mediators involved, including growth factors, extracellular vesicles, soluble lipid mediators, cytokines, and chemokines. Understanding these areas will ultimately identify potential cells, mediators, and interactions for therapeutic targeting.

## 1. Epithelial Roles in Tissue Repair

**Figure 1:**
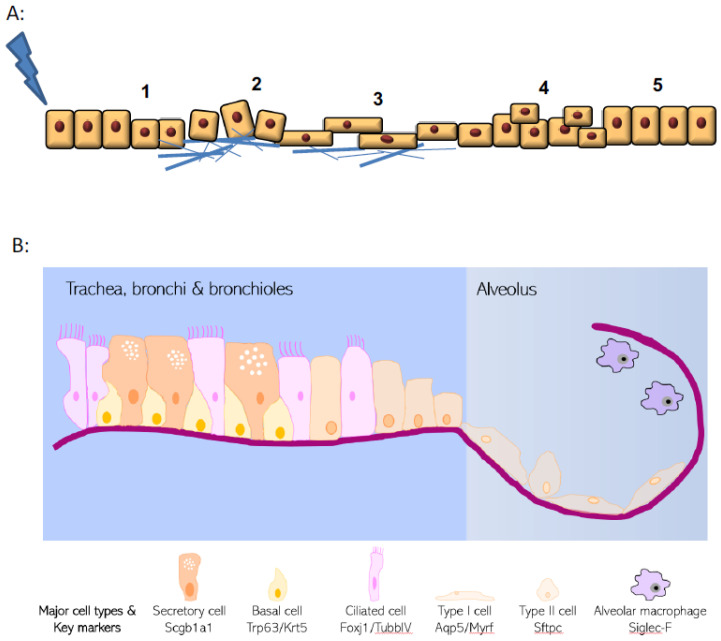
Hallmarks of wound healing and pulmonary epithelial organisation. (**A**) Upon injury, epithelial cells undergo a multistage process to repair damage. These phases include i. dedifferentiation from specialised and mature cells, ii. adhesion to extracellular matrix, iii. spreading and migration towards the wound site, iv. cellular proliferation and finally v. redifferentiation and repair. (**B**) Structure of the pulmonary epithelium and organisation of major epithelial cell types.

Lungs are continually exposed to infections, toxins, and airborne pollutants that stress homeostasis. Consequently, respiratory disorders cause a vast burden of global disease and are among the leading causes of death worldwide [1]. Although a quiescent organ at baseline the lungs have a significant reparative capacity in response to injury [2] (Figure 1A), but dysregulated inflammation and aberrant or defective repair mechanisms are increasingly linked to the pathobiology of several diseases including COPD, asthma pulmonary fibrosis and acute respiratory distress syndrome (ARDS) [3]. Given that the pulmonary epithelium is central to host defence, homeostasis, and disease biology, this review highlights the role of airway epithelium in repair, with a particular focus on the mediators involved. While not an exhaustive assessment of the current literature, this review will focus on the interaction and interplay of epithelial regeneration and inflammatory processes.

## 2. Epithelial Structure and Evolving Knowledge on Progenitor Populations

The lung epithelial cellular structure and composition varies significantly along its proximal–distal axis (Figure 1B). Within the trachea and conducting airways, the epithelium is arranged predominantly as a pseudostratified layer, with the most frequent cell types being ciliated cells, secretory cells, and basal cells that are adherent to the basal lamina [4]. In addition, small numbers of neuroendocrine cells and tuft cells are also present. This is in distinct contrast to the alveolar regions where thin type I cells (AT1) lie in close apposition to endothelial cells for efficient gas exchange, along with the presence of cuboidal type II cells (AT2) that produce pulmonary surfactant proteins. The three main cell types within the pulmonary epithelium that have well-documented progenitor potential are basal cells, secretory cells, and the AT2 cells [5]. However, these cells are unusual in that they display remarkable plasticity and heterogeneity in response to injury. Pulmonary progenitor cells are frequently fully differentiated epithelial cells with specialised function rather than populations of immature precursor cells. New approaches to identifying and phenotyping epithelial cells (such as single cell sequencing approaches) are continuing to reveal additional complexity and heterogeneity to respiratory epithelial cell identity [6,7,8].

**Figure 2:**
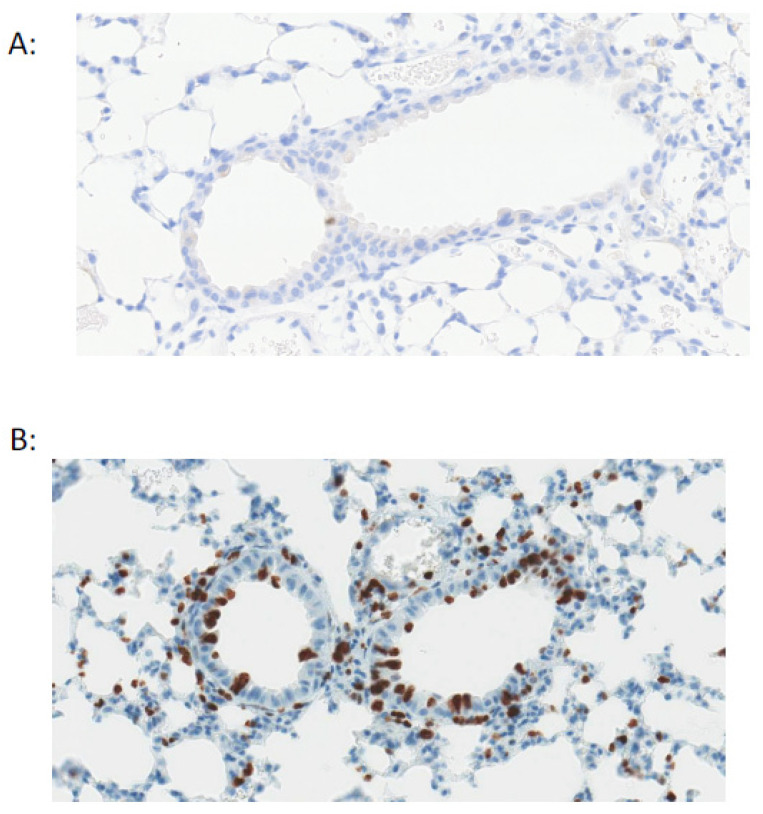
Pulmonary epithelial cells are quiescent during homeostasis. Staining for Ki67^+ve^ cells (brown) shows very low proliferation in the mouse airway under basal conditions (**A**) with an increase in proliferation after airway epithelial injury with naphthalene (**B**).

Original studies using pulsed thymidine [9] have been complemented by recent lineage tracing studies that together show the pulmonary epithelium is quiescent during homeostasis with very low rates of turnover (Figure 2). However, in response to injury, the epithelium can mount a robust response with many cells re-entering the cell cycle to divide and/or differentiate or dedifferentiate [2]. Within the trachea and proximal airways basal cells appear to be the predominant progenitor population [10]. Basal cells are characterised by expression of Trp63, cytokeratin Krt5, integrin α_6_, podoplanin, and nerve growth factor receptor (p75), and are present in the trachea and most of the conducting airways in human lungs (albeit in declining numbers more distally) [2]. By contrast, these cells are only present in more proximal airways in mice. They proliferate in response to epithelial injury and are capable of self-renewal and differentiation into secretory and ciliated cells [10]. They can also restore denuded tracheal xenografts implanted into immunodeficient mice [11].

In distal airway epithelium, a more prominent role for secretory cells as the major progenitor population has been described, although it should be noted that rodent lungs lack basal cells in this region, in contrast to the small numbers of basal cells that also populate this region in humans. Secretory cells are characterised by expression of the secretoglobins Scgb1a1 and Scgb3a2. Under homeostatic conditions, distal airway secretory cells self-renew and differentiate into ciliated cells [12] (which are characterised by the expression of the transcription factor Foxj1 and the cytoskeletal component Tubb4a). Secretory cell injury is most frequently modelled by systemic administration of naphthalene which targets cytochrome p450 (Cyp2f2)-expressing secretory cells. In response to naphthalene-induced injury, those secretory cells that survive (Cyp2f2 negative variant club cells) proliferate and reconstitute both secretory and ciliated cells [12,13]. Furthermore, secretory cells are capable of mediating tracheal epithelial repair, albeit making a minor contribution in the context of sulfur dioxide (SO_2_)-induced injury [12]. In addition, after marked basal cell injury (induced by a Krt5–diphtheria toxin system) secretory cells are capable of dedifferentiating and repopulating the basal cell population with similar self-renewal rates as ‘normal’ basal cells, with these secretory cell-derived basal cells also able to promote epithelial repair and give rise to all three main epithelial cell types after influenza infection [14].

Within the alveolar region, the best described progenitor cell is the AT2 cell, which proliferates after injury and can repopulate AT1 cells [15,16]. However, other candidate alveolar progenitor cell populations exist. These include a lineage negative progenitor that subsequently expresses Trp63 and Krt5 (traditional basal cell markers) and is able to migrate into the alveoli to repair areas of severe damage after influenza injury [17,18], an α_6_β_4_ integrin-expressing cell type that proliferates in response to bleomycin-induced alveolar injury and proliferates and expands clonally in ex vivo culture [19], and a H2-K1 high distal airway epithelial population that can differentiate into alveolar structures [20]. In addition, a population of cells at the bronchoalveolar duct junction (BADJ) that co-expresses both AT2 and secretory markers (surfactant protein C and Scgb1a1, respectively) has been reported to have both alveolar and airway progenitor potential [21]. Given that some AT2s co-express these markers, confirmation of this cell type as a bona fide and independent progenitor population is awaited.

In summary, these studies demonstrate that while the respiratory epithelium has specific region-defined cellular modes of repair, significant plasticity exists in the cells that are able to respond to injury, and that injury-specific and magnitude of injury-specific regenerative process may be invoked. While the cellular players in epithelial reconstitution are increasingly delineated, much work is needed to define the local microenvironmental cues that accelerate healing.

## 3. Epithelial Cell–Immune Cell Crosstalk

**Figure 3:**
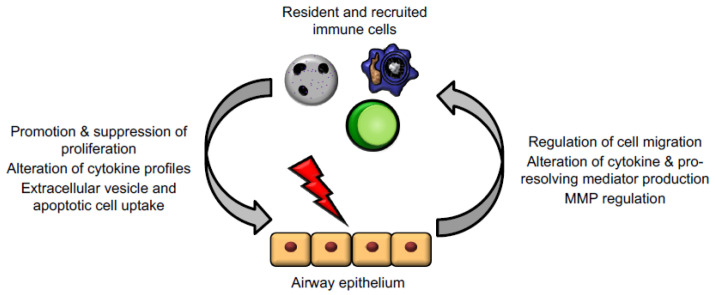
Epithelial cells and immune cells engage bidirectional communication to regulate tissue repair. Activated immune cells, including macrophages, neutrophils, and T lymphocytes, regulate epithelial cell responses to promote proliferation, alteration of mediator and cytokine production, and downstream signalling cascades. In turn, injured epithelium can themselves regulate immune cell trafficking and promote a shift from inflammation to resolution and repair functions.

The crosstalk between epithelial cells and immune cells (particularly granulocytes and macrophages) is critical for the appropriate progression of inflammation, resolution, and repair in the lung (Figure 3). As well as being important barrier cells segregating organs from potential hostile environments, epithelial cells are important for regulating immune cell trafficking, triggering changes in mediator and cytokine production, altering phagocytosis during inflammation resolution, and changing production of proteins/MMP to allow for epithelial repair. Epithelium and immune cells can also work together for the generation of certain signals, like specialised pro-resolving mediators (SPMs) which require transcellular synthesis [22]. The role of immune cells in resolution and repair (and some of the ways in which epithelial cells promote these functions) have been extensively reviewed elsewhere.

Immune cells, in turn, are responsible for helping to lay down scaffolding proteins, promoting epithelial migration and cellular survival and preventing a shift from beneficial repair to fibrotic processes. Neutrophils act as the first responder in inflammation and help neutralise the wound area of infection, helping to promote early stages of inflammation. Regarding the later steps of tissue repair, neutrophils serve as major producers of reactive oxygen species (ROS), nitric oxide (NO), TGF-β, and other mediators that promote epithelial cell migration and proliferation. There is also evidence that infiltration of neutrophils into mucosal epithelium (within the intestine) triggers increased epithelial permeability [23]. It is important to recognise that a balance of neutrophil numbers and activation is key as prolonged neutrophil influx can potentially impair tissue repair, perhaps by maintaining a pro-inflammatory mediator milieu or impairing a shift to repair phenotypes in macrophages and epithelial cells.

Macrophages have a more established role in tissue repair, though the precise molecular mechanisms of action for these cells in tissue repair are still largely unknown. A role for macrophages in tissue repair was first suggested nearly 50 years ago, and multiple mouse models with depleted macrophages (or impaired cellular migration) have impaired wound healing [24,25,26]. Often, macrophages have a critical, temporally restricted role in tissue healing. This is elegantly highlighted in salamander limb repair after amputation, whereby macrophage presence around the initial time of injury is essential for regeneration of the amputated limb. Furthermore, the defective limb repair observed in macrophage-depleted states can be rescued by the combination of allowing macrophage replenishment and then causing additional amputational injury [24]. Some of the roles of macrophages are the same as neutrophils—production of mediators, cytokines, and growth factors that promote epithelial cell proliferation; regulation of oxidative stress; and modulation of epithelial barrier properties—but distinct macrophage roles also exist. For one, macrophages remain a major phagocytic cell, and clearance of debris, wounded epithelial cell fragments, and apoptotic neutrophils is critical for promoting a space for repair and for triggering production of many downstream cellular signals. Indeed, macrophages (along with cough and the mucociliary escalator) is an important part of airway clearance within the healthy lung. Generally, tissue-resident macrophages and recruited monocytes (often differentiated by cytokines and growth factors in the local tissue microenvironment) significantly contribute to tissue repair, regeneration, and the mechanisms of fibrosis, highlighting their substantial plasticity [27]. IFN-γ- or LPS-stimulated macrophages are instrumental in the initial stages, where phagocytosis aids in pathogen killing and clearing debris, whereas IL-4-treated macrophages support angiogenesis and matrix production in the later stages of wound healing [27,28]. The unique roles at the different repair stages have been reviewed elsewhere [29].

Within lung, alveolar macrophages are the most widely studied, and are major regulators of matrix metalloproteinases (MMPs), which are critical for epithelial cell migration [30,31]. Lung epithelial cells, as well as macrophages, are also capable of producing IL-10, with alveolar macrophages having high expression of the IL-10 receptor, which acts to limit inflammatory responses at least partly via JAK1–STAT3 pathways [32,33]. Finally, there is evidence that macrophages may promote matrix deposition and provide scaffolding, which would allow for better epithelial cell migration and re-epithelialisation. In a mouse model investigating the effect of recruited macrophages to the site of skin repair after mechanical injury, peritoneal and tissue-resident macrophages in the skin, spleen, and liver in LysMCre/iDTR mice were depleted at the various stages of healing. Depletion in the inflammatory phase (2 and 1 days prior to wounding as well as at day 2 and 4 post-wounding) resulted in delayed re-epithelialisation and reduced collagen formation. By contrast, depletion of macrophages in the tissue formation phase (mid-stage of the repair response 3, 4, 6, and 8 dpi) was shown to delay wound closure and lead to haemorrhage in the wound tissue [34,35,36]. Within the lung, a second population of tissue resident macrophages also exists, namely interstitial macrophages, with both similarities and differences when compared to alveolar macrophages; their function remains relatively poorly described [37].

It is important to note that dysregulated macrophage function, such as incomplete efferocytosis, can contribute to fibrosis and improper wound healing as well as autoimmune diseases such as systemic lupus erythematous and type I diabetes [38]. For example, in the case of type I diabetes, which occurs as a result of the destruction of insulin producing B cells in the pancreas, aberrant efferocytosis of apoptotic pancreatic cells leading to necrosis is thought to contribute to the release of autoantigens. In addition, impaired efferocytosis is seen in a multitude of diseases, including diabetes and asthma, with evidence that efficient apoptotic cell sensing and clearance is critical for efficient tissue repair [39]. For example, slow wound healing in diabetes is associated with accumulated apoptotic cells at the wound site [38]. Therefore, critical to a healthy repair process is the control of macrophage function and signals that disrupt normal healing and lead to fibrotic scar generation by epithelium and macrophages.

Like the innate immune system, epithelial cells can trigger and respond to adaptive immune cells. Damaged cells relay signals to natural killer (NK) cells, T cells, and innate lymphoid cells (ILCs), among others. For example, the cytokines IL-25 and IL-33 produced by epithelial cells induce Th2-type adaptive responses where increased expression of both cytokines has been found in patients with idiopathic pulmonary fibrosis (IPF) [40,41,42], and IL-33 has an inhibitory effect on mast cell functions [43]. Lymphocytes can directly respond to these signals, trafficking into the injured space, or can relay these signals on to other cell types [44]. Furthermore, certain T cell populations (γδ T cells) can reside in the intraepithelial spaces; these can provide epithelial growth factors and help regulate epithelial cell apoptosis [45]. Depletion of adaptive immune cells leads to more severe lung injury, for example, regulatory T cells promote tissue repair by promoting Th1 and Th17 cell responses [46]. The direct correlation of depleted T cell populations (and NK cells) to tissue repair seen in other organ systems (like the skin) has not yet been established [45,47]. Lymphocytes are also major producers of cytokines, including IL-22 (a major driver of epithelial cell proliferation and repair), IL-4/IL-13 (which may regulate the balance between epithelial cell healing and fibrosis), and amphiregulin (an EGF family member which has been linked to tissue repair and remodelling) [48,49,50]. While cytokine production and the contribution of adaptive immune cells to dysregulated healing is well characterised, the contributions of these cells in regular pulmonary epithelial healing are less clear. Generally, both innate and adaptive immune cells appear to have highly regulated and wide-ranging roles in regulating and responding to epithelial injury; further investigation into the signals that mediate these responses may reveal significant novel targets to promote repair.

## 4. Infection Influence on Tissue Repair

Infection by both viral and bacterial pathogens is a very frequent cause of lung epithelial injury while pathogen presence can further impede the repair process by causing additional tissue damage and by prolonging the effects of pro-inflammatory cytokines [51]. Furthermore, inflammatory cells recruited to injured sites and bacterial endotoxins contribute to destruction of the ECM by overexpression of matrix metalloproteases [52]. Nowhere have the consequences of pathogen-induced pulmonary epithelial injury been more apparent than the SARS-CoV-2 pandemic, where diffuse alveolar damage (DAD, the hallmark of acute respiratory distress syndrome) is a frequent finding in fatal disease [53]. Furthermore, SARS-CoV-2-triggered inflammation leads to additional virus-independent immunopathology with treatment by anti-inflammatory corticosteroids able to reduce mortality in severe disease [54]. In studies using the Gram-negative bacteria *Pseudomonas aeruginosa* in airway epithelial models, infection was found to inhibit cell proliferation and alter directional cell migration during the repair process [55]. The main *Pseudomonas aeruginosa* secreted virulence factors (e.g., ExoA and LecB) are thought to enact their effects through the induction of reactive oxygen species, ERK/p38 (MAPK) signalling and increased NF-κB transcriptional activity [56,57]. Vitamin D, a steroid hormone known to have anti-inflammatory properties, has been suggested as a prognosticator and potential therapeutic target for pulmonary fibrosis and viral infections [58]. Vitamin D supplementation was found to prevent bleomycin-induced lung fibrosis in a murine model which supported previous studies showing that deficiency exacerbated fibrosis through activation of the renin−angiotensin system and promotion of the TGF-β/SMAD signalling pathway [58,59]. Vitamin D deficiency is associated with increased risks of pulmonary viral infection [60], with data suggesting that vitamin D may enhance type I interferon responses, endogenous mediators of antiviral immunity.

## 5. Mechanisms for Promoting Tissue Repair

**Figure 4:**
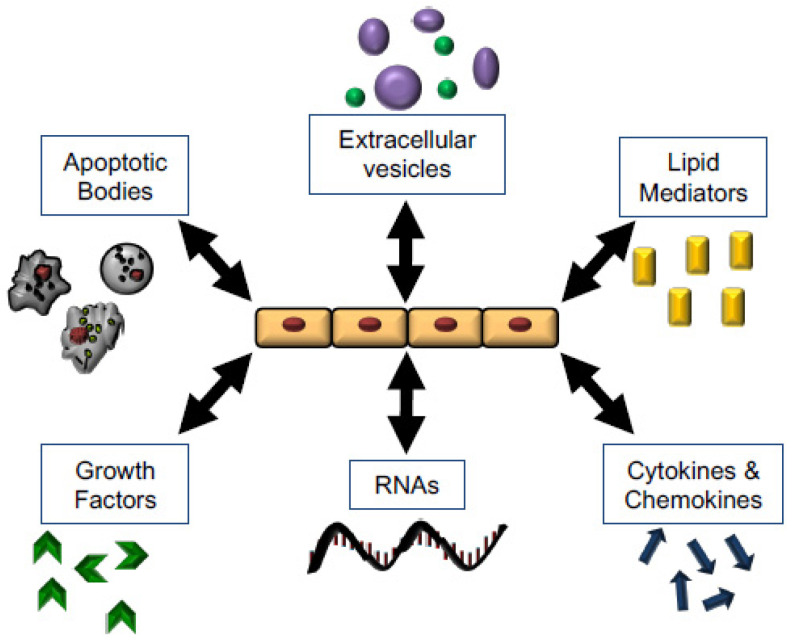
Mechanisms of Wound Healing and opportunities for therapeutic intervention. Epithelial cells regulate and respond to multiple stimuli which have the potential to mediate and promote tissue repair, including apoptotic bodies, microvesicles, lipid mediators, soluble signals, RNAs and miRNAs, and growth factors.

Key to the promotion of wound repair and resolution is cell–cell communication. Here, we expand on some of the mechanisms of communication used by immune and parenchymal cells to promote wound healing effects by epithelial cells (Figure 4 and Table 1).

### 5.1. Growth Factors

The major established mediators which affect epithelial cells in tissue repair are growth factors, including epidermal growth factor (EGF), insulin growth factor (IGF), vascular endothelial growth factor (VEGF), and transforming growth factors (TGFs). The link between EGF, EGF receptor (EGFR), and epithelial cell proliferation/repair is well established, with early observations that EGFR was increased in epithelial cells after injury and correlated with increased epithelial proliferation [61,62]. Many different inflammatory stimuli stimulate EGFR phosphorylation and activation, including endotoxin, cadmium, dual oxidase-1, house dust mite, naphthalene, and more. The downstream effects of increased EGFR activation include short-term changes in epithelial cell–cell contacts and reduced epithelial cell barrier resistance, increased epithelial cell migration, epithelial cell proliferation, activation of integrin pathways, and smaller wound areas (which could be a result of either migration or proliferation or both). Evidence exists for EGF being a major ligand to activate EGFR in these repair processes, although several other ligands can also activate EGFR [63,64,65,66]. More directly, EGF treatment of epithelial cells promoted faster epithelial cellular proliferation, migration, and wound healing, demonstrably through phosphorylation of, and signalling by, EGFR [67,68,69,70]. EGF may promote tissue repair by multiple mechanisms, but EGF can stimulate translocation of scaffolding proteins to the cell membrane, providing evidence for a role of EGF in early repair processes [71].

Insulin growth factor (IGF) signalling is emerging as another major regulator of epithelial growth and regeneration. IGF-1 and -2 are both expressed throughout gestation, mainly in the mesodermal-derived components of the respiratory tract, and at the same time as the proliferation of adjacent epithelial cells [72]. In adult human lungs, IGF-1 has been detected in interstitial macrophages, alveolar macrophages, and epithelial cells of IPF patients, despite being primarily present in interstitial macrophages in non-IPF controls. A higher ratio of IGF-1^+ve^ macrophages (compared to all interstitial macrophages) correlated with collagen and disease severity [72,73]. IGF-1 is also increased in mouse airways and primarily in epithelial cells after LPS exposure which, in turn, leads to increased anti-apoptotic proteins (Bcl-2) and apoptosis resistance in these cells [74]. Similarly, IGF-1 and IGF-1R are elevated after 48 h of hyperoxia exposure, and are mainly expressed in the alveolar and airway epithelium. Hyperoxia also stimulated increased cellular proliferation, which was moderately reduced by the use of anti-IGF-1 antibodies [75]. Recent studies have begun to further elucidate the effects of IGF-1 on epithelial cell responses. Using an LPS-induced lung injury mouse model, alveolar epithelium was found to increase alveolar macrophage production of IGF-1 through TGF-β. This resulted in decreased IL-1β, TNF, and MCP-1 production and promoted epithelial phagocytosis of apoptotic cells to promote the resolution of airway inflammation and accelerate the repair of inflammatory injury [76]. Ghosh et al. also demonstrated that IGF-1 secretion is increased after scratch wounding of epithelial cells [77]. Scratch wounding and IGF-1 both stimulated WNT expression alongside differentiation of type II epithelial cells to type I, suggesting that IGF-1 may also be affecting epithelial cell fate [77]. Overall, the evidence for IGF-1-stimulated proliferation in other organs [78] and the potential roles for IGF-1 in the lung suggest an untapped area of research and call for further investigations into the role of IGF-1 in lung epithelial cell-centred wound repair.

Several other growth factors also play roles, including VEGF, TGF, and TGF-β. Varet et al. demonstrated that VEGF stimulated proliferation of alveolar type II cells [79], and Roberts et al. demonstrated that VEGF stimulated faster wound closer and proliferation following scratching in vitro [80]. However, assessment of protein expression of VEGF and VEGFxxxb isoforms in tissue using immunohistochemistry and ELISA in BAL of ARDS patient samples showed decreased expression compared to healthy samples [79,81,82]. A murine model of LPS-induced ALI and lung-targeted ablation of the VEGF gene in VEGFloxP mice also found no increase in the expression of VEGF in epithelial cells post-infection and no decrease in alveolar cell proliferation was detected by Western blot in the VEGF knockout mouse [81,83]. By contrast, TGF-β—which shares 42% sequence homology with EGF, can stimulate EGFr, and colocalises in the same areas of the airways (including bronchiolar and alveolar epithelium)—has similar effects as EGF in that it promotes faster wound healing [84,85]. Finally, TGF-β is a major player in multiple lung processes, and is increased upon epithelial cell wounding; however, there is far more evidence for TGF-β as a pro-fibrotic signal that derails normal repair processes rather than stimulating them, largely through its promotion of epithelial–mesenchymal transitions [86,87,88]. As research into epithelial repair processes continues, growth factors still remain the largest category of established players, both for promoting repair (EGF, IGF-1) and for potentially sabotaging it (TGF-β).

### 5.2. Soluble Lipid Mediators

Epithelial cells can also communicate via production of and responsiveness to lipid mediators, including prostaglandins, leukotrienes, and specialised pro-resolving mediators (SPMs) via transcellular synthesis [89,90]. Prostaglandins (PGs) are lipid mediators derived from arachidonic acid via enzymatic production. They can have both pro- and anti-inflammatory roles, with the most well-studied phenomenon being the contribution of PGE_2_ to pain signalling as one of the cardinal signs of inflammation. A large body of work has focused on the role of PGs in modulating epithelial cell and fibroblast crosstalk, with particular emphasis on fibrotic remodelling. These studies have been previously reviewed, but it is worth reiterating the tight regulation of PG production in this context, underscoring the importance of epithelial cells in controlling the appropriate balance of repair [91].

PGE_2_ is constitutively produced by epithelial cells and can be released from dying cells [92], and can stimulate cellular proliferation and wound closure; blockade of prostaglandin production also impairs epithelial cell wound healing [93,94,95]. Interestingly, the optimal concentrations of PGE_2_ may be cell dependent, as Savla et al. showed that higher concentrations were better for 16HBE cells and lower concentrations were better for normal human bronchial epithelial cells (NHBEs) in enhancing wound closure [95]. Furthermore, PGE_2_ was equally effective when given at the same time as wounding or 2 h later, but had reduced efficacy when given 4 or 6 h post-wounding. This, combined with evidence from studies outside the lung, suggests PGE_2_ plays a role in early wound healing processes, though it did not appear to affect cellular migration [94,95]. Given the wide breadth of action for this particular lipid mediator, and the lack of effect shown with other PGs and leukotrienes, further investigations into the timing and regulation of PGE_2_ in the context of proper wound healing may bear important results.

SPMs are endogenously produced lipid mediators derived from omega-3 and omega-6 fatty acids with multiple classes, including lipoxins (Lxs), resolvins (Rvs), and maresins (MaR) [96,97]. The broad-ranging capabilities of these mediators have been seen in the lung and epithelial cells (among other organs and cell types) [22]. A growing body of work has investigated SPMs in promotion of wound healing, particularly regarding epithelial cells, as they can produce SPMs and express the receptors that they signal through [98,99]. LxA_4_ treatment of primary human alveolar type II epithelial cells and bronchial cells increased wound healing and epithelial cell proliferation [100,101,102]. Similarly, in an acid-injury model in mice, RvD3 promoted increased epithelial cell proliferation and wound closure, contributing to faster resolution and healing [103]. LxA_4_ also assists in late-stage repair by restoring normal epithelial cell functions, namely tight junctions, liquid surface tension, and lung compliance (MaR1 also restored tight junctions and normal lung permeability) [101,104,105,106,107]. Lastly, several studies show that SPMs (including RvD1, LxA_4_, and MaR1) promote appropriate wound healing by preventing a shift to fibrosis. This largely occurs through prevention of epithelial–mesenchymal transition, as marked by a reduction in fibronectin and α-smooth muscle actin, a restoration of E-cadherin to normal levels, and prevention of morphological change [105,108,109]. This is coupled with prevention of fibroblast proliferation, demonstrating that the effects on proliferation are cell specific [100]. While data for the contribution of these lipid mediators are still emerging, their roles in inflammation resolution and epithelial function suggest that they are also important regulators of wound healing.

### 5.3. Cytokines

Several cytokines traditionally thought of as being primarily associated with inflammatory responses have also been shown to directly influence epithelial functions and repair. Chemokine receptor-3 (CCR3) ligands (CCL11, CCL24, and CCL26) accelerate epithelial wound closure in vitro, with epithelial CCR3 expression upregulated in human asthma [110]. IL-4 and IL-13, classically associated with a Th2 allergic response, also influence migration and proliferation of primary airway epithelium, but their roles on lung repair in vivo require further exploration. IL-22 has been demonstrated to promote airway epithelial repair in vivo, with IL-22-deficient mice having reduced epithelial proliferation and exacerbated collagen deposition and morbidity in the context of influenza infection [111,112]. Conventional NK cells were found to be the predominant source of IL-22 during influenza infection, with adoptive transfer of IL-22 sufficient NK cells into IL-22 deficient mice able to partly rescue the impaired epithelial healing seen in these mice [112]. It seems likely that increasing numbers of inflammatory mediators will subsequently be recognised as having roles in tissue regeneration, and is an area of investigation that will likely lead to further leads for pro-reparative strategies.

### 5.4. RNA, Apoptotic Bodies, Microvesicles, and Exosomes

A number of microRNAs have been identified as having altered expression profiles in regenerating lungs after influenza injury [113]. These include miR-290, miR-21, let-7, and miR-200, which are predicted to target genes involved in repair, and direct testing of their functions in regenerating epithelium is underway. For example, miR-21 works by silencing signalling molecules involved in NF-κB-induced inflammation and TNF expression, and has been shown to be upregulated in macrophages after efficient efferocytosis, thus suppressing TNF and inducing IL-10 [114]. A number of microRNA clusters have also been associated with development of the distal airways (e.g., miR-17-92 and the miR302/367 cluster [115,116], with manipulation of these noncoding RNAs altering epithelial proliferation and differentiation. Whether such developmentally restricted RNAs can be ‘reactivated’ during injury in the airway epithelium is an exciting avenue of research.

The transfer of cellular contents and signals via extracellular vesicles (including apoptotic bodies, microvesicles, and exosomes) is another means by which cell–cell communication can occur, and can serve as a means of RNA shuttling between cells. To briefly review these classes, apoptotic bodies are formed during apoptosis and are typically >1 μm in diameter. Microvesicles (MVs), also known as extracellular vesicles or microparticles, are small vesicles that result from plasma membrane budding. They have a diameter in the range of ~100 nm–1 μm and carry biologically active cargo which they can deliver to recipient cells. MVs have great heterogeneity and numerous biological roles; details about their packaging, transfer, and effects in acute lung injury have been reviewed elsewhere [117,118]. Exosomes are typically 30–100 nm in size and derived from an endocytic origin. Importantly, all categories of extracellular vesicles can be taken up by recipient cells through phagocytic processes and can deliver their contents without additional processing. These characteristics make them ideal messengers, and underscore why they are of growing interest in cell biology.

The process of cell death by apoptosis is a frequent outcome of injury, and is known to induce compensatory proliferation in epithelial beds termed apoptosis-induced proliferation (AiP) [119]. AiP is dependent upon production of reactive oxygen species and is amplified by immune cell recruitment in drosophila [120], but the role and mechanisms of AiP in lung epithelial repair remains understudied. Whether apoptotic bodies are involved in AiP, and the role of apoptotic bodies in lung epithelial repair, await study. Given that macrophage-derived apoptotic bodies can transfer microRNA-221/222 to induce proliferation in lung epithelium in vitro [121], the role of apoptotic bodies in lung repair after injury is worthy of further investigation.

Microvesicles from multiple origins can be taken up by epithelial cells, and have been shown to promote proliferation and repair in corneal and renal wounds [122,123,124]. These tubular renal epithelial cells (and lung airway epithelial cells) can also produce exosomes and MVs themselves, which can influence other cells involved in the repair process (such as fibroblasts) or promote inflammatory functions in immune cells [125,126,127]. While the majority of research regarding MVs in the lung has focused on their usefulness as biomarkers or their effects on immune cells in inflammatory processes, these same methods of cellular communication could be present during the repair process. From the few studies that have been conducted, we do know that the cellular origin MVs plays an important role in modulating cellular responses. For instance, MVs from T cells inhibited cell growth and promoted apoptosis of recipient 16HBE cells, but exosomes from bone marrow MSCs promoted epithelial cell proliferation [124,128]. Interestingly, in the MSC study by Tomasoni et al., the MVs and exosomes from MSCs contained IGF-1 receptor and were no longer effective when IGF-1R had been silenced [124]. Furthermore, despite the fact that both dermal fibroblasts and BM-MSCs contain IGF-1R, only the exosomes from BM-MSCs contained this receptor, which suggests specificity in packaging mechanisms. This study highlights that the role for MVs and exosomes in wound repair (as well as other processes) may be critically dependent upon cargo selection. Overall, there is growing evidence that all three categories of extracellular vesicles are important for cell–cell communication with investigations studying their role in resolution and repair processes urgently needed. As one of the main potential cargoes of extracellular vesicles is RNA (including both message RNA and small RNA species), the potential exists for responding inflammatory cells to directly influence the transcriptome of injured epithelium, thereby influencing tissue regeneration [121].

### 5.5. Secondary Messengers

ATP released by injured epithelial cells binds to purinergic receptors (ligand-gated P2X receptors and G-coupled P2Y receptors) and triggers a Ca^2+^ wave as well as G-protein-coupled receptor activation, leading to downstream EGFR activation and related signalling pathways [129]. Remodelling the cytoskeleton, regulated by members of the Rho family, is a key factor for cellular adhesion and migration, allowing for successful wound healing. Cyclic adenosine monophosphate (cAMP), an intracellular secondary messenger whose formation is catalysed from ATP by adenylate cyclases enzymes and is implicated in several downstream signalling pathways activates cAMP-dependent protein kinase A I (PKA). PKA activation has been shown to promote cellular migration in bronchial epithelial cells by activation of A_2A_ receptors, thereby accelerating wound closure [130]. Intracellular cAMP concentration can be increased by agonist binding to adrenergic receptors (ARs), specifically β-ARs, which are commonly used in patients with pulmonary disease [131].

**Table 1:** Summary of soluble mediators implicated in epithelial repair and fibrosis.

Mediator	Effects on Repair	Implication in Fibrosis	Key References
Growth factors			
EGF	EGF and its receptor upregulated after airway injury. Promotes migration and wound healing of primary airway epithelial cells in vitro. EGF receptor dominant negative mutant impair basal cell proliferation after injury in vivo.	Overexpression of EGF receptor in bronchial epithelium and type 2 pneumocytes of IPF patients. EGFR inhibition by gefitinib results in development of pulmonary fibrosis.	[62,64,66,132,133]
IGF	Increases expression of anti-apoptotic proteins in airway epithelial cells. Also associated with increased ECM deposition and fibrosis.	Increased IGF-1 present in IPF tissue and associated with decreased pulmonary function and disease progression. Inhibition of IGF-1R by OSI-906 delayed progression and decreased mortality in murine lung.	[73,74,134]
VEGF	Alveolar cell proliferation and enhanced wound healing in vitro	VEGF-A from AT2 cells may play protective role and aid regeneration of wall defects. VEGF-Axxxa family is profibrotic and VEGF-Axxxb is inhibitory.	[79,80,135,136]
TGFα	Increased wound healing of alveolar cells in vitro.	Chronic conditional expression of TGFα induces pulmonary fibrosis independently of inflammation in adult murine lung.	[85,137]
Lipid mediators			
PGE_2_	Enhanced proliferation and wound closure of airway epithelium in vitro.	Inhibition of the PGE_2_ degrading enzyme, 15-Prostaglandin dehydrogenase, increases PGE_2_ concentrations and ameliorates lung function and increases proliferation in a bleomycin mouse model of pulmonary fibrosis. Potent downregulator of fibroblast activation.	[94,95,138,139]
Lipoxin A_4_	Promotes primary alveolar epithelium proliferation and wound closure, inhibits apoptosis and cytokine production in vitro.	Decreased lipoxin A4/LTB_4_ ratio advances fibrosis. Upregulation of ALX receptor associated with reduced collagen accumulation in vivo.	[100,101,139]
RvD3	Increased epithelial proliferation and reduced inflammation and organ injury after acid-induced lung injury in vivo.		[103]
Cytokines			
CCR3 ligands	Upregulated epithelial proliferation and chemotaxis and enhanced wound repair in vitro.	Lung fibrotic response limited by neutralising CCR3 receptor, expression of profibrotic mediators decreased.	[110,140]
IL-22	Promotes airway epithelial proliferation and protects against lung dysfunction, morbidity, and fibrosis after influenza infection in vivo.	Protective role against severe fibrosis following bacterial infection.	[111,112]
Other			
Airway mucin gene (*MUC5B*)	Attenuates ciliated cell differentiation in repair. MUC5B disrupts alveolar repair by interfering with the interaction between AT2 and the matrix.	Promoter polymorphism is a strong genetic risk for IPF.	[141,142]

## 6. Looking Forward: What Lies Next?

Although there is understanding and characterisation of the key mechanisms that govern tissue homeostasis and repair following lung injury, it is evident that much is still to be discovered. Whether the soluble mediators, signalling cascades, and cellular regenerative responses characterised in murine models are translatable to human disease largely remains to be determined. The process of augmenting the resolution of inflammation as a potential therapeutic strategy is increasingly being established within the literature; accelerating epithelial repair after injury and inflammation may well provide another complementary approach to addressing unmet clinical needs.

## Data Availability

Not applicable.
